# Baseline immune profile by CyTOF can predict response to an investigational adjuvanted vaccine in elderly adults

**DOI:** 10.1186/s12967-018-1528-1

**Published:** 2018-06-05

**Authors:** Christine M. D. Lingblom, Sangeeta Kowli, Nithya Swaminathan, Holden T. Maecker, Stacie L. Lambert

**Affiliations:** 1 Department of Immunity, Transplant, Infection, Stanford University, Stanford, USA; 2 Translational Sciences, MedImmune, Mountain View, CA USA

**Keywords:** RSV, Vaccine, CyTOF, Immune profile

## Abstract

**Background:**

Mass cytometry, or CyTOF (Cytometry by Time-of-Flight), permits the simultaneous detection of over 40 phenotypic and functional immune markers in individual cells without the issues of spectral overlap seen in traditional flow cytometry.

**Methods:**

In this study, we applied CyTOF to comprehensively characterize the circulating immune cell populations in elderly individuals both before and after administration of an investigational adjuvanted protein vaccine against respiratory syncytial virus (RSV) in a Phase 1a trial. Antigen-specific T cell responses to RSV by IFNγ ELISPOT had been observed in most but not all recipients in the highest dose cohort in this trial. Here, CyTOF was used to characterize the cellular response profile of ELISPOT responders and non-responders in this vaccine dose cohort.

**Results:**

Both CD4^+^ and CD8^+^ T cell antigen-specific IFNγ responses were observed. Principal components analysis revealed baseline differences between responders and non-responders, including differences in activated (HLA-DR^+^) CD4^+^ and CD8^+^ T cells, which were higher in non-responders versus responders. Using viSNE to analyze RSV-responsive CD4^+^ and CD8^+^ T cells, we also found increased expression of HLA-DR, CCR7, CD127 and CD69 in non-responders versus responders.

**Conclusions:**

High parameter CyTOF can help profile immune components associated with differential vaccine responsiveness.

**Electronic supplementary material:**

The online version of this article (10.1186/s12967-018-1528-1) contains supplementary material, which is available to authorized users.

## Background

Aged adults have decreased immune responses compared to younger adults and are more prone to acute infections as well as reactivation of latent viruses. Waning adaptive immunity can be seen in adults as young as 50 years old [1]. Extensive research on immune senescence in the elderly has identified multiple pathways by which aging mechanisms adversely affect immune responses, particularly T cell responses [2–4]. Increased background inflammation, decreased antigen presenting cell function, a higher threshold of T cell activation, decreased naïve T cell numbers, a loss of T cell receptor diversity, a loss of central memory CD8 T cells and reduced CD8 T cell priming are all mechanisms identified that impact T cell responses in older adults [5–7].

T cells in combination with neutralizing antibodies may have a key role in controlling respiratory viruses such as influenza and respiratory syncytial virus (RSV) that can cause more acute infections in the elderly versus healthy young adults. In an elderly adult population, T cell IFNγ responses to influenza could distinguish between those protected by vaccination and those who subsequently developed influenza illness [8]. Pre-existing influenza-specific CD4^+^ T cells were associated with decreased illness severity following influenza challenge of healthy volunteers lacking neutralizing antibodies [9]. Though neutralizing antibody titers to RSV are similar between elderly and young adult populations [10], the elderly have decreased RSV-specific T cell responses compared to young adults [11, 12].

Vaccines have been only partially successful in reversing declining immune responses in older adults. Those meant for elderly subjects may benefit from inclusion of an adjuvant [13–15] or an increased antigen dose [16, 17]. There is currently no approved vaccine for RSV though the incidence of RSV illness in older adults is on par with that of influenza illness [18]. It has been proposed that a successful vaccine for the elderly would need to induce both protective neutralizing antibodies and virus clearing T cells [19, 20].

An investigational adjuvanted RSV vaccine that aimed to induce both neutralizing antibodies and virus-specific T cells was evaluated in dose-finding Phase 1 trials in > 60 year old subjects [21]. This vaccine consists of RSV soluble fusion protein formulated without or with the adjuvant Glucopyranosyl Lipid A in 2% stable emulsion (GLA-SE). Humoral and cellular responses were measured in > 60 year olds following vaccine dosing. At the 80 ug RSV sF + 2.5 μg GLA-SE dose tested, 100% of recipients demonstrated a > threefold rise in humoral responses and 74% demonstrated a > threefold rise in cellular responses [21]. A higher dose of 120 μg RSV sF + 5 μg GLA-SE induced similar rates of humoral and slightly higher rates of cellular immune responses [22]. The goal of the work described here was to use a targeted multiparameter evaluation of the RSV F-specific T cell response to further characterize the cellular response to RSV in these vaccinated subjects.

To more comprehensively characterize T cell responses to RSV, we used CyTOF mass cytometry, a highly multiparametric version of flow cytometry that uses heavy metal ion labels and mass spectrometry as the readout in lieu of fluorochromes and light detection. This methodology has the dual benefit of allowing many more specificities to be probed in parallel in the same samples, while dramatically reducing spillover between detector channels, which is a major issue in fluorescence flow cytometry [23]. Using stimulation with RSV F antigen peptides, or with Phorbol 12-myristate 13-acetate (PMA) + ionomycin, we were able to read out antigen-specific as well as global immune parameters using CyTOF, and to relate these to vaccine response as measured by enzyme-linked immune spot (ELISPOT).

## Methods

### Human samples

Heparinized whole blood was collected from 20 healthy older adults (60 years and older) with informed consent under an institutional review board-approved, randomized phase 1a study of MEDI7510 (NCT02115815). Demographics for this cohort are presented in Table 1. Clinical endpoints, F-specific antibody, and F-specific IFNgamma ELISPOT responses have been previously reported [21]. Samples were taken pre-vaccination, D8 and D29 post-vaccination from those subjects dosed with 80 μg of soluble RSV fusion protein sF adjuvanted with Glucopyranosyl Lipid A in 2% stable emulsion (GLA-SE) (Immune Design Corporation, USA). Peripheral blood mononuclear cells (PBMC) were separated by Histopaque Ficoll within 6 h of blood draw and cryopreserved in serum-free CTL Cryo™ ABC (CTL, USA), then transferred to a central lab for LN_2_ storage until testing.

**Table 1:** Demographics of study cohort

Demographic parameter	Non-responder (5)	Responder (14)
Age (year; average± SD)	70 ± 7	70 ± 7
Age (year; range)	62–79	61–82
Female [number, (%)]	4, (80%)	6, (43%)

### IFNgamma ELISPOT

Cryopreserved PBMC from study subjects were batched by subject, thawed in CTL Wash medium (Cellular Technology, USA) with benzonase nuclease (Novagen-Millipore USA), washed and resuspended in CTL Test medium (Cellular Technology, USA), and tested by F-specific IFNgamma ELISPOT as previously described [24]. Briefly, viable cells were plated at 300,000 cells/well in quadruplicate in human IFNgamma ELISPOT plates (Mabtech, USA) and stimulated with either medium containing 0.1% dimethyl sulfoxide (DMSO) (mock) or 2 μg/mL of overlapping peptide pools of RSV F (JPT GmbH, Berlin, Germany). 30,000 viable cells/well were stimulated with *Staphylococcus aureus* enterotoxin B (SEB) as a positive control. At 20–24 h plates were developed and counted on the ImmunoSpot Analyzer (Cellular Technology, USA). Data was expressed as the spot forming cells (SFC) per million PBMC after background subtraction of mock wells, with a lower limit of detection of 33 SFC/million PBMC (Additional file 1).

### Extracellular and intracellular staining and CyTOF analysis

PBMC from study subjects as well as a healthy control sample for each batch of samples were thawed in warm CTL 10× wash medium (CTL, USA) diluted 1:10 in RPMI (Gibco-Life Technologies, USA) containing l-Glutamine (Gibco-Life Technologies, USA) and Benzonase nuclease (Novagen-Millipore, USA), washed twice then resuspended in CTL test medium (CTL, USA) containing l-Glutamine, and viable cells were counted by Vicell (Merck Millipore, USA). Cells were added to a V-bottom microtiter polystyrene plate at 1 million viable cells/well, for each sample one well was kept as unstimulated, one for the RSV F peptide pool stimulation (overlapping 15-mers custom-produced by JPT, final concentration 5 μg/mL, [24]) for 6 h and one for PMA/Ionomycin (Sigma-Aldrich, USA, final concentration 10 ng/mL and 1 μg/mL, respectively) for 4 h at 37 °C, in a CO_2_ incubator. Simultaneously, activation reagent, Brefeldin A (Sigma-Aldrich, USA), and secretion inhibitor Monensin (Sigma-Aldrich, USA) was added to all the wells. PMA, Ionomycin, Brefeldin A and Monensin was diluted in CyPBS (10× PBS without heavy metal contaminants diluted 1:10 in MilliQ water, ROCKLAND, USA). Final DMSO and ethanol concentration from all sources (peptides, brefeldin A, monensin) did not exceed 0.5%. At the end of stimulation, 0.8 μL 5 M EDTA was added to the wells, to a final concentration of 2 mM and incubated for 15 min at room temperature. The cells were washed three times with CyFACS (CyPBS with 2 mM EDTA and 0.05% sodium azide) followed by extracellular staining for 45 min on ice with 70 μL of the antibody cocktail (Table 2). All antibodies were either from purified unconjugated, carrier-protein-free stocks from eBiosciences, Biolegend, or R&D Systems that we conjugated with metal isotopes ourselves or they were conjugated with metal isotopes from Fluidigm. The cells were washed three times with CyFACS buffer and then resuspended in 100 μL CyPBS of 1:3000 diluted 5 mg/mL Live-Dead (1,4,7,10-tetraazacyclododecane-1,4,7,10-tetraacetic acid (DOTA)-maleimide containing natural-abundance indium 115, Macrocyclics, USA) and incubated 30 min on ice. The cells were washed three times with CyPBS and then resuspended in 100 μL 2% para-formaldehyde (PFA) in CyPBS and placed at 4 °C overnight. The next day the cells were washed three times with eBioscience permeabilization buffer (1× in MilliQ water) followed by intracellular staining for 45 min on ice with 70 μL of the antibody cocktail (Table 2) before washing three times with CyPBS. The cells were resuspended in 100 μL iridium-containing DNA intercalator (1:2000 dilution in 2% PFA in CyPBS; Fluidigm) and incubated at room temperature for 20 min. The cells were washed three times in CyPBS and three times in MilliQ water. The cells were diluted in a total volume of 700 μL in MilliQ water before injection into the CyTOF Helios™ (Fluidigm). The data were normalized using Normalizer v0.2 MCR [25] (Additional file 2). Data analysis was performed using FlowJo v10 by gating on intact cells based on the iridium isotopes from the DNA intercalator, then on singlets by DNA intercalator versus event length, then on live cells which is the Indium-Live-Dead negative population, followed by cell subset gating (Additional file 2, Fig. 2a).

**Table 2:** Antibody panel for CyTOF ICS, with metal labels, clones and source

No.	Specificity	Metal label	Clone	Source
1	Live/dead	In115	–	In house
2	CD49d	Pr141	9F10	Fluidgm
3	CD19	Nd142	HIB19	Fluidgm
4	ICOS	Nd143	DX29	Fluidgm
5	CD69	Nd144	FN50	Fluidgm
6	CD4	Nd145	RPA-T4	Fluidgm
7	CD8	Nd146	SK1	Fluidgm
8	CD20	Sm147	2H7	Fluidgm
9	CD57	Nd148	HCD57	In house
10	CD54	Sm149	HA58	In house
11	CD134 (OX-40)	Nd150	ACT35	Fluidgm
12	CD107a	Eu151	H4A3	Fluidgm
13	TNFα	Sm152	Mab11	Fluidgm
14	CD45RA	Eu153	HI100	Fluidgm
15	CD3	Sm154	UCHT1	Fluidgm
16	CD28	Gd155	L283	In house
17	CD38	Gd156	HB-7	In house
18	HLA-DR	Gd157	G46-6	In house
19	CD33	Gd158	WM53	Fluidgm
20	CD11c	Td159	Bu15	Fluidgm
21	CD14	Gd160	M5E2	Fluidgm
22	IFNγ	Dy161	4S.B4	In house
23	CD80	Dy162	2D10.4	Fluidgm
24	IL-4	Dy163	MP4-25D2	Fluidgm
25	IL-17	Dy164	N49-653	Fluidgm
26	CD127	Ho165	A019D5	Fluidgm
27	IL-2	Er166	MQ1-17H12	Fluidgm
28	CD27	Er167	L128	Fluidgm
29	CD40L	Er168	24–31	Fluidgm
30	CCR7	Tm169	15053	In house
31	PD1	Er170	EH12.1	In house
32	Granzyme B	Yb171	GB11	Fluidgm
33	NKG2C	Yb172	134591	In house
34	CD25	Yb173	M-A251	In house
35	CD16	Yb174	3G8	In house
36	Perforin	Lu175	B-D48	Fluidgm
37	CD56	Yb176	NCAM16.2	Fluidgm
38	DNA1	Ir191	–	Fluidgm
39	DNA2	Ir193	–	Fluidgm
40	CD11b	Bi209	ICRF44	Fluidgm

### Statistical analyses

#### Multivariate analyses

Multivariate analyses of pattern recognition “orthogonal projections to latent structures by means of partial least squares discriminant analysis” (OPLS-DA) were performed using the SIMCA-P (version 14.1) statistical package (MKS Data Analytics Solutions, Malmö, Sweden). OPLS-DA is a development of principal component analysis (PCA), in which Y variables are introduced and their relationship to X variables examined. In our case, multivariate models were created where study patients were set as Y variables (Group Y1 for non-responders and group Y2 for responders) and 29 components (cytokine responses and phenotype markers) were set as X variables. In the figures the 13 variables with biggest impact on the models are shown. The two-component models (PC1 and PC2) is defined by a value for explanatory power or goodness of fit, R2, which estimates the amount of variance in Y that is explained by the X-variables. A high value indicates that the selected X-variables have generated a model that can explain differences that exist between the studied groups. A model is also given a value for stability, Q2, which describes the validity of the model. This is determined with cross validation, a procedure where one study subject is removed and the capacity of the remaining subjects to predict the separation between the groups is assessed. This procedure is repeated for all the subjects; a high value indicates that the model is stable no matter which subject is excluded. A number between 0 and 1 is given or 0–100%. 0 being worst and 1 being best. pq1 is a value that explains the impact that the X variables has on the model. The program is set to mean centering and unit variance scaling to give all variables an equal chance of providing model leverage independently of data scale and distribution.

#### viSNE (visual high-dimensional single-cell data analysis based on the t-Distributed Stochastic Neighbor Embedding (t-SNE) algorithm)

viSNE is a dimensionality reduction algorithm that permits visualization of multi-dimensional data as a two dimensional scatter plot. We performed viSNE analysis in cytobank (Cytobank, Santa Clara, CA). Boolean ‘OR’ gates for CD107a, IFNγ, TNFα and IL-4 from responders (n = 14) and non-responders (n = 5) were concatenated in FlowJo v10.1 for both CD4^+^ and CD8^+^ T cells after RSV (F) peptide stimulation at day 0 and day 8. For comparative analysis, samples were down sampled and viSNE maps were generated from a mixture of equal-sized samples (CD4^+^ T cells = 30,205 events per sample; CD8^+^ T cells = 10,437 events per sample). The event count for each T cell population was determined by the sample with the lowest events. After importing the concatenated files into cytobank, viSNE was run using default cytobank parameters (iterations = 1000, perplexity = 30 and theta = 0.5). In each figure, all samples were derived from the same viSNE run. viSNE maps show median marker expression for each population. Scales on the maps are individually generated for each marker with the intensity levels from low (blue) to high (red) expression.

#### Univariate analyses

Unpaired *t* test was used to determine statistical significance between the study subjects on day 0 and day 8 as well as on day 0 and day 29. GraphPad Prism 7.0 was used to plot graphs (GraphPad, San Diego, CA, USA).

## Results

### T cell responses by IFNγ ELISPOT

**Fig. 1:**
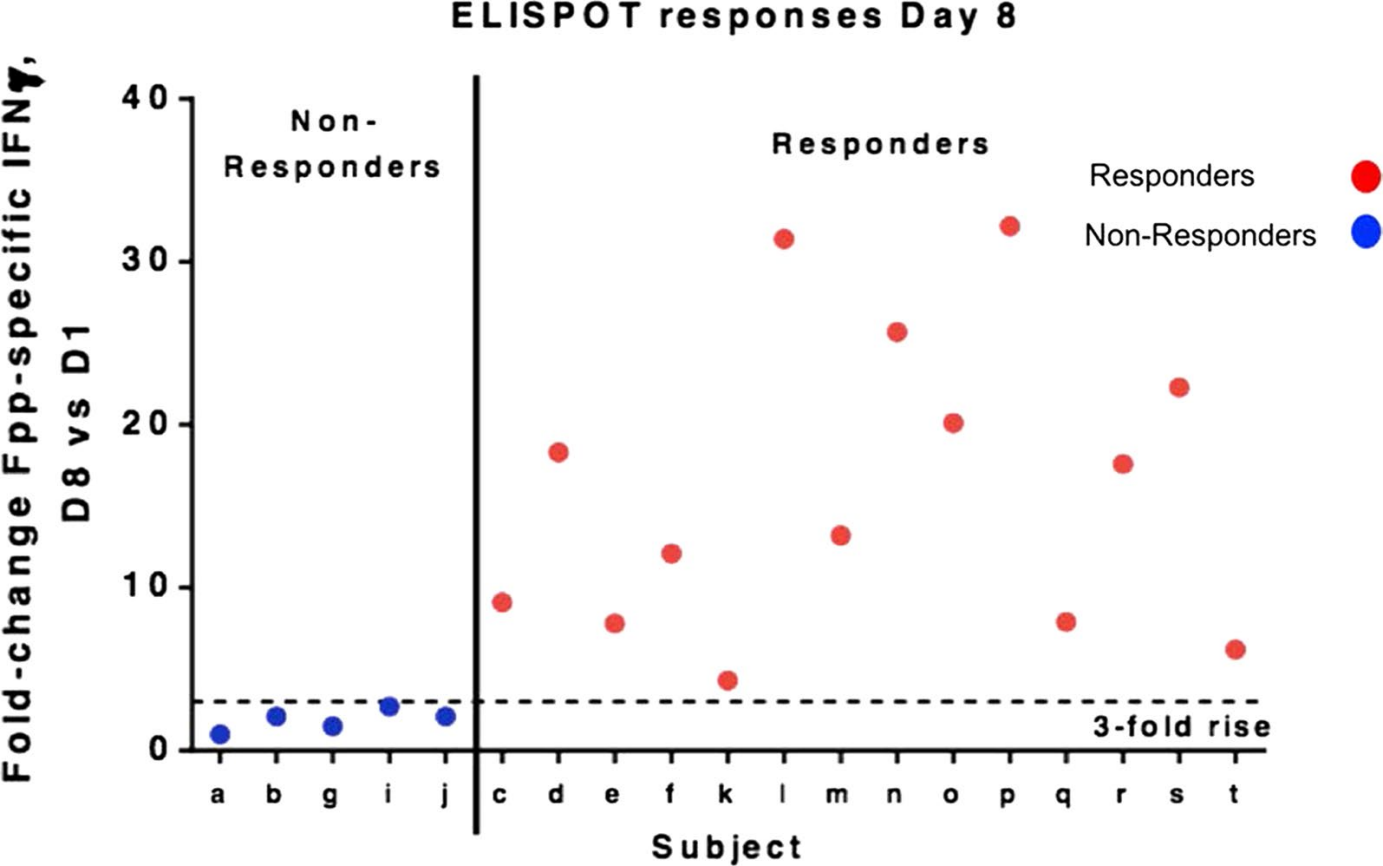
IFNγ^+^ ELISPOT responses, Day 8 vs Day 1. A threefold rise was declared as a responder

As previously reported, pre- and post-vaccination PBMC obtained from the clinical trial participants were tested for T cell responses by a qualified F-specific IFNγ ELISPOT assay, with a peak response at Day 8 post vaccination [21]. A minimum threefold change in F-specific responses at Day 8 versus pre-vaccination was used to designate responders by this assay. Among the 20 subjects in the treatment cohort, one subject was dropped as the PBMC viability was low and the sample failed the acceptance quality criteria for the F-specific IFNγ ELISPOT assay. Of the 19 subjects with reportable data for both the prevaccination and Day 8 timepoints, 14 subjects demonstrated a ≥ threefold rise in responses, with responses ranging from 4.3- to 32.2-fold over baseline (Fig. 1). 5 subjects with a < threefold rise in responses were designated as non-responders. These ELISPOT responses were used to categorize the vaccine subjects for subsequent multiparameter intracellular cytokine staining by CyTOF analysis. Cytomegalovirus (CMV) status is unknown for the study subjects; CMV may affect T cell response rates to other antigens.

### CyTOF analysis and detection of RSV-specific T cell responses

**Fig. 2:**
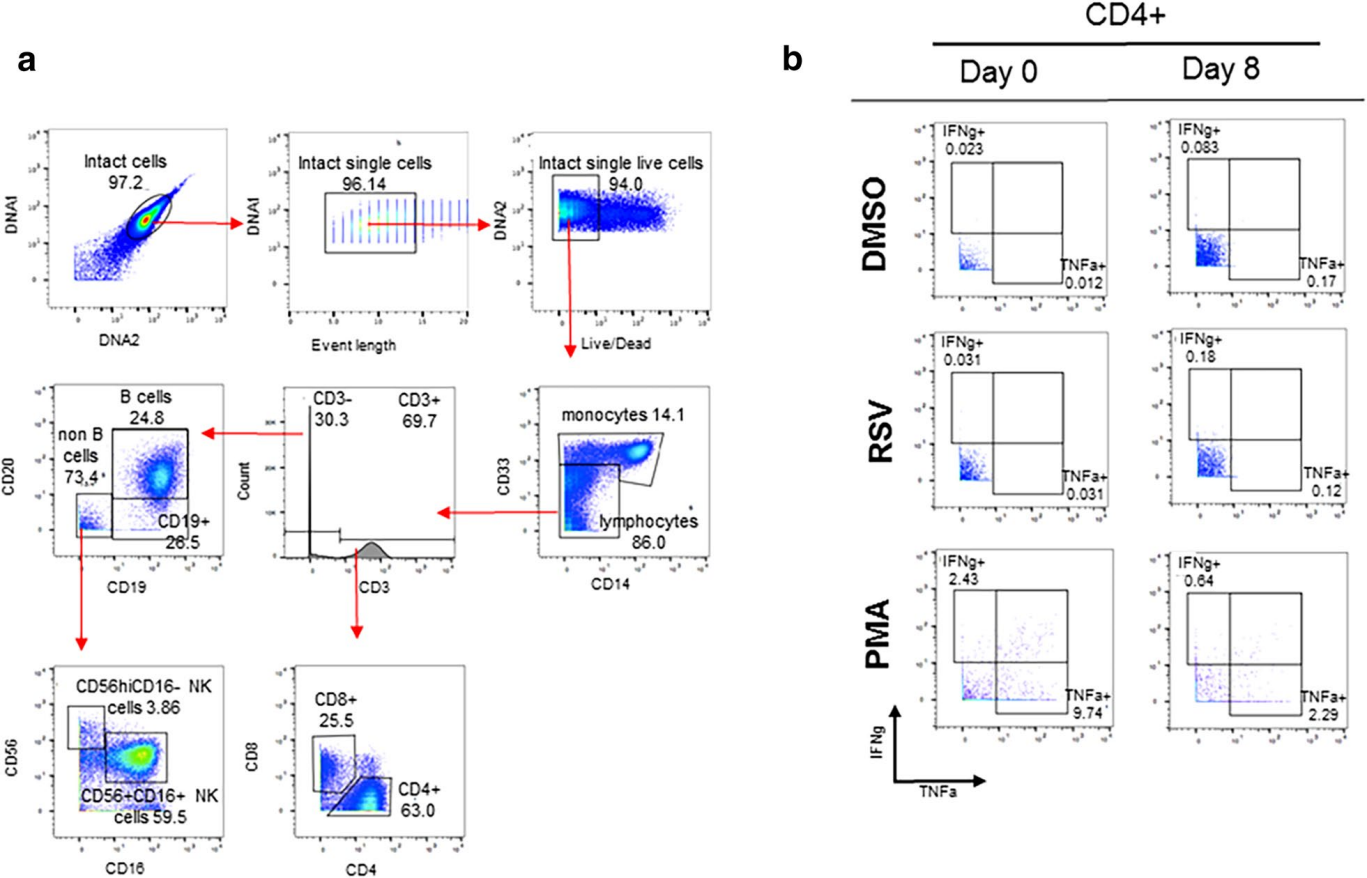
CyTOF gating hierarchy of peripheral blood mononuclear cells (PBMCs). Major immune cell subsets including monocytes, CD4^+^ and CD8^+^ T cells, B cells and natural killer cells were identified with a manual gating strategy post-normalization using FlowJo v10.1 software (**a**). Representative dot plots of CD4^+^ T cell cytokine (TNFα^+^ and IFNγ^+^) responses at day 0 and day 8 (**b**)

A representative manual gating schema used to identify major peripheral blood subsets including B cells, CD4^+^ and CD8^+^ T cells, NK cells and monocytes is shown in Fig. 2a. While the percentage of CD3^+^CD4^+^ T cells was slightly higher in the non-responders compared to the responders (Additional file 3A), no significant differences were found in either the CD3^+^CD4^+^ or CD3^+^CD8^+^ T cell percentages between responders and non-responders, pre-vaccine or on day 8 or 29 post-vaccine (Additional file 3A, B). Figure 2b is a representative dot plot showing the induction of CD4^+^ IFNγ^+^ and CD4^+^ TNFα^+^ after stimulation with RSV s(F) pp on D8.

### Th1 versus Th2 responses

**Fig. 3:**
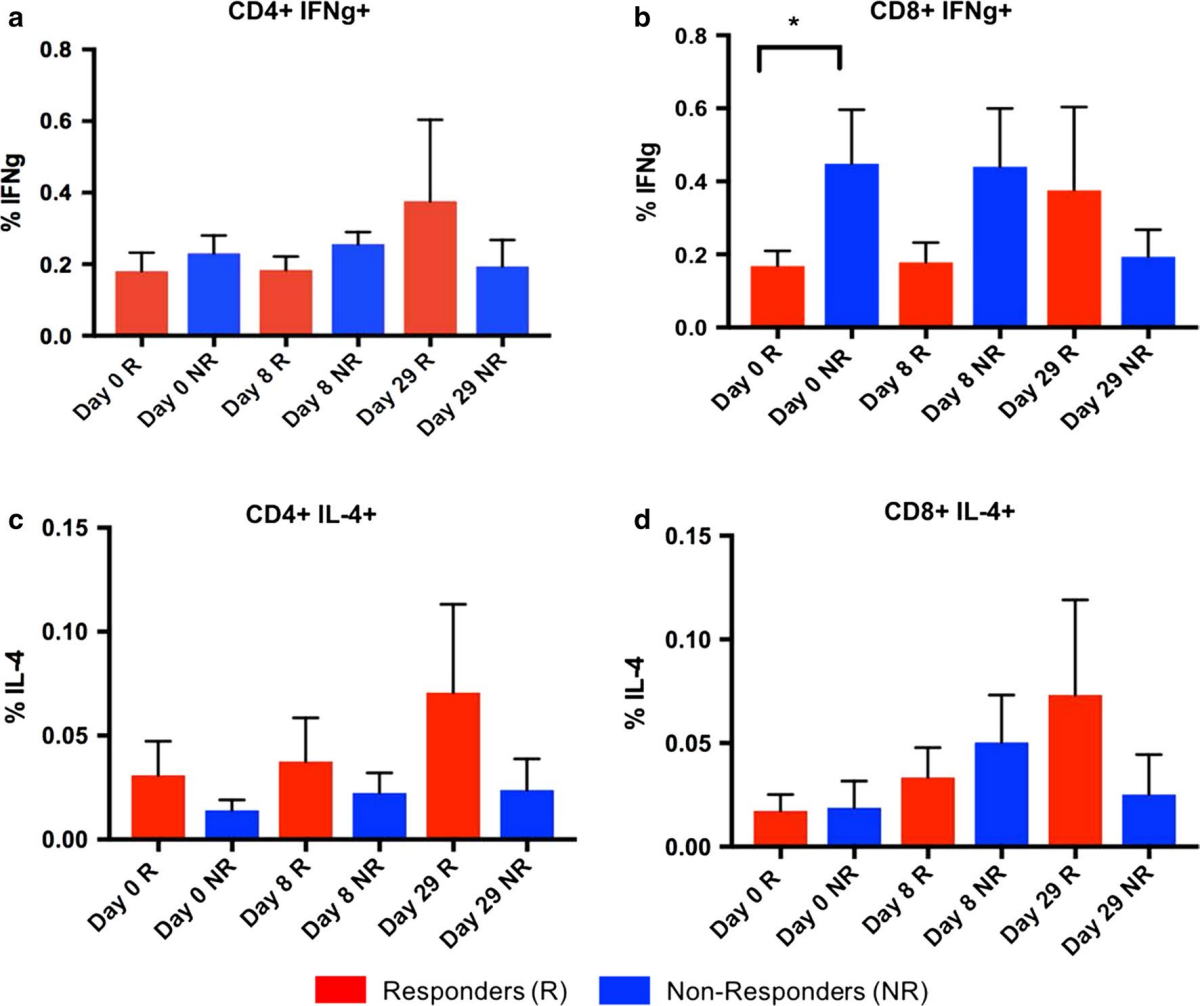
IFNγ expression in CD4^+^ and CD8^+^ T cells. Responders develop CD4^+^ T cell IFNγ^+^ response over time after stimulation with RSV (F) peptides. IFNγ^+^ responses in CD8^+^ T cells shows significant differences (p < 0.05) between responders (n = 14) and non-responders (n = 5) at day 0 after stimulation with RSV (F) peptides (**a**, **b**). Responders develop CD4^+^ T cell and CD8^+^ T cell IL-4 responses over time (**c**, **d**)

To determine the effects of RSV s(F) pp on Th1 and Th2 responses between responders and non-responders, levels of IFNγ and IL-4 were independently assessed by manual gating in FlowJo. RSV s(F) pp stimulation resulted in both CD4^+^ and CD8^+^ IFNγ responses, and CD4^+^ IL-4 responses (Fig. 3a–c). An upward trend was observed in the responders for both CD4^+^ and CD8^+^ IFNγ^+^ and CD4^+^ IL-4^+^ at D8 and D29 compared to D0. Furthermore, we observed significantly higher baseline levels of CD8^+^ IFNγ in the non-responders compared to the responders at D0 (before vaccination) (Fig. 3b). A gradual increase in the frequency of IL-4^+^ producing CD8^+^ T cells post-stimulation with RSV s(F) pp was also seen in the responders (Fig. 3d).

### Longitudinal patterns of cytokine responses

**Fig. 4:**
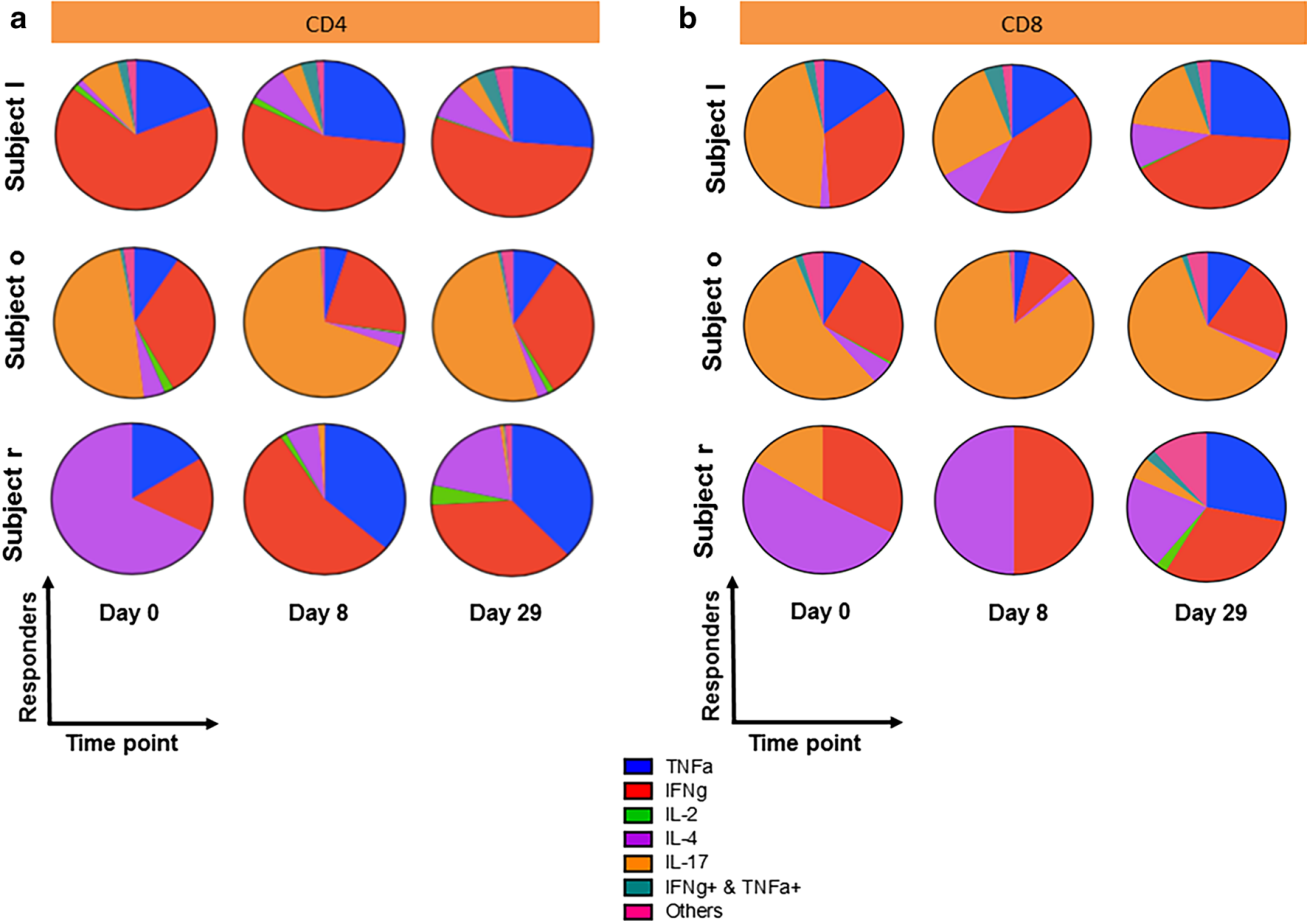
Multifunctional analysis of CD4^+^ and CD8^+^ T cell cytokine responses to RSV. Day 8 and day 29 boolean ‘OR’ gates for CD4^+^ and CD8^+^ T cell IFNγ^+^, TNFα^+^, IL-2^+^, IL-4^+^ and IL-17^+^ expression in response to RSV (F) peptide stimulation, were generated in Flowjo 10.1. The average of the boolean ‘OR’ gates was used to determine the top three responders. Multifunctional analysis revealed heterogeneity in cytokine production among the subjects as well as dominance of single cytokine production both pre- and post-vaccination compared to dual- or tri-CD4^+^ (**a**) and CD8^+^ (**b**) positive cytokines

To evaluate the multifunctionality of CD4^+^ and CD8^+^ T cells in response to RSV, we assessed the expression of TNFα^+^, IFNγ^+^, IL-2^+^, IL-4^+^, IL-17^+^ as well as dual- and tri-cytokine positive combinations pre-(day 0) and post-vaccination (day 8 and day 29). Shown here are the top three responders. The average of day 8 and day 29 boolean ‘OR’ gates for IFNγ^+^, TNFα^+^, IL-2^+^, IL-4^+^, IL-17^+^ expression was used to determine the top three responders. In subject l, IFNγ^+^ expression is dominant at baseline. Post-vaccination on day 8 and day 29, TNFα^+^ expression as well as the proportion of dual IFNγ^+^ TNFα^+^ CD4^+^ T cells is markedly upregulated (Fig. 4a, top panel). In subject o, the proportion of IL-17^+^ CD4^+^ T cells is dominant pre- and post-vaccination. We also find that the expression of TNFα^+^ and IFNγ^+^ CD4^+^ T cells reduces slightly post-vaccination on day 8 compared to pre-vaccination (day 0) but increases post-vaccination on day 29 to levels comparable to pre-vaccination (day 0) (Fig. 4a, middle panel). In subject r, pre-vaccination (day 0) the proportion of IL-4^+^ CD4^+^ T cells is most dominant. However, post-vaccination on day 8 and day 29 we observe a switch from IL-4^+^ CD4^+^ T cells to TNFα^+^ and IFNγ^+^ CD4^+^ T cells (Fig. 4a, bottom panel). In CD8^+^ T cells, post-vaccination in subject l, the cytokine production switches from IL-17^+^ (day 0) primarily to IFNγ^+^ at day 8 and day 29 (Fig. 4b, top panel). In subject o, IL-17^+^ production is dominant both pre-(day 0) and post-vaccination (day 8 and day 29) (Fig. 4b, middle panel). In subject r, post-vaccination (day 29), induced TNFα^+^ CD8^+^ T cells as well as dual- and tri-cytokine positive CD8^+^ T cells (Fig. 4b, bottom panel). These results suggest that first, there is substantial heterogeneity in cytokine production among the subjects. Second, pre- and post-vaccination, single cytokine production is dominant compared to the contribution from dual- or tri-positive cytokines both in CD4^+^ and CD8^+^ T cells. In general, the proportion of the single cytokine population prevalent for any given subject pre-vaccination (day 0) does not dramatically change post-vaccination (day 8 and day 29).

### Principal components analysis of RSV-specific T cell responses

**Fig. 5:**
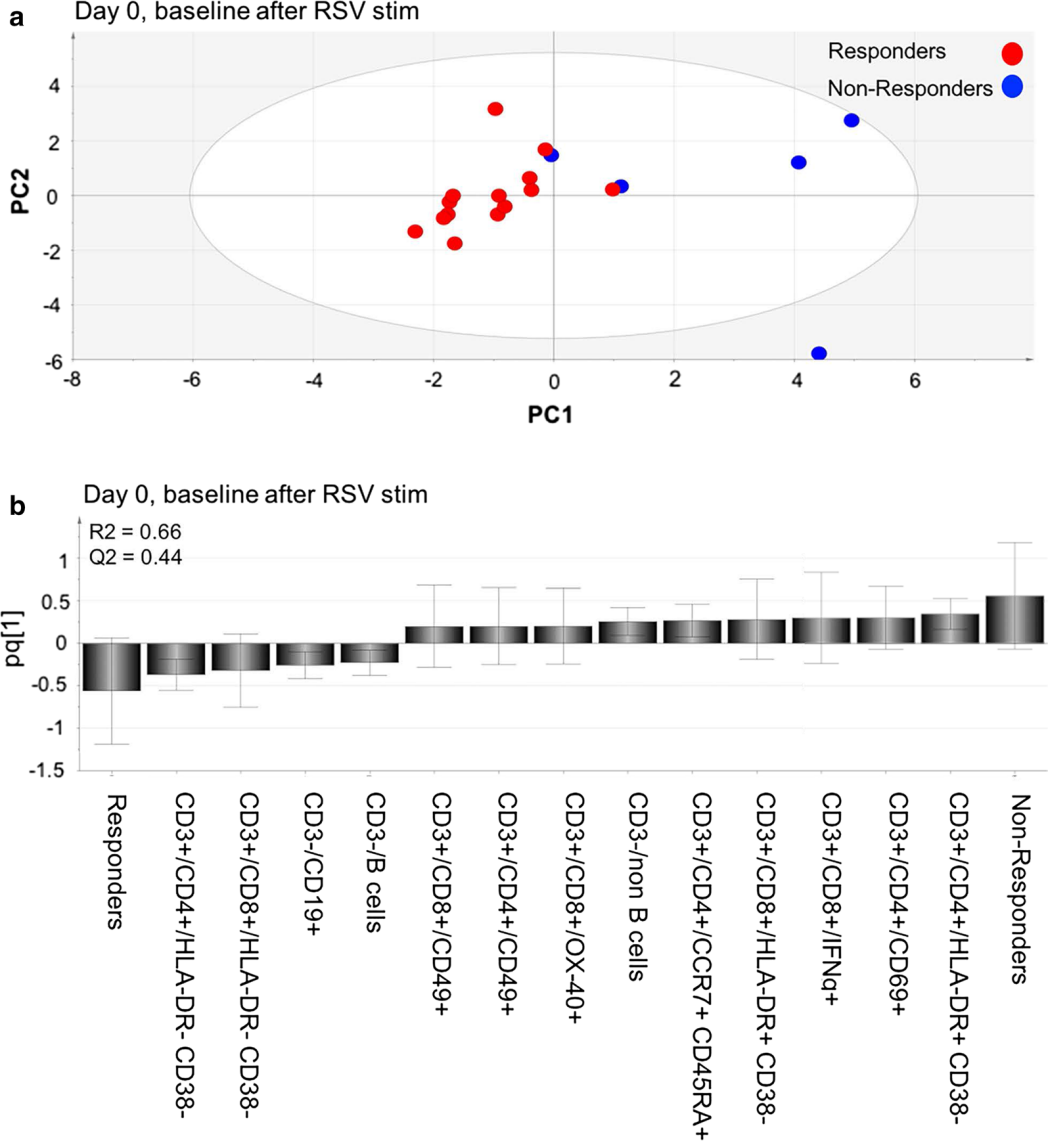
PCA analysis on gated subsets. **a** Multivariate analysis of cytokine responses and phenotype marker levels in the study groups at day 0, before vaccination. “Orthogonal partial least square-discriminant analysis” (OPLS-DA) was done to see if the cytokine responses and markers (X-variables) could segregate the two study groups (Y-variables, e.g. non-responders (n = 5) and responders (n = 14) before vaccine against RSV was given. The generated model had a stability of 44% (Q2Y = 0.44) and explanatory power of 66% (a goodness of fit R2Y = 0.66). **b** Column graph of the OPLS-DA was done to see which variables had the largest impact on the separation of the two groups (responders and non-responders at day 0). Variables closest to the subject group are positively associated

The multivariate method of OPLS-DA was used to see if it was possible to predict the outcome of the vaccine based on the immune profile of the patients at baseline. We divided the patients into two groups, responders (R) and non-responders (NR), based on the results from the IFNγ ELISPOT (Fig. 1). The cytokine responses as well as surface marker levels after RSV stimulation at baseline (day 0) were set as study variables (X) and the two study groups were set as outcome variables (Y). The two study groups formed two distinct clusters, a tight one composed of responders and a scattered one composed of non-responders (Fig. 5a). When we constructed a loading plot to see which variables that contributed to the separation, interestingly, the non-responders were positively associated with CD4^+^HLA-DR^+^CD38^−^ and CD8^+^HLA-DR^+^CD38^−^ and the responders with CD4^+^HLA-DR^−^CD38^−^ and CD8^+^HLA-DR^−^CD38^−^. We also found that non-responders had higher levels of CD4^+^ CD69^+^, CD8^+^ IFNγ^+^ and non-B-cells at baseline and responders had higher levels of CD3^−^CD19^+^ and B cells (Fig. 5b). The generated model had a stability of 44% (Q2Y = 0.44) and explanatory power of 66% (a goodness of fit R2Y = 0.66).

### viSNE analysis of RSV-specific T cell responses

**Fig. 6:**
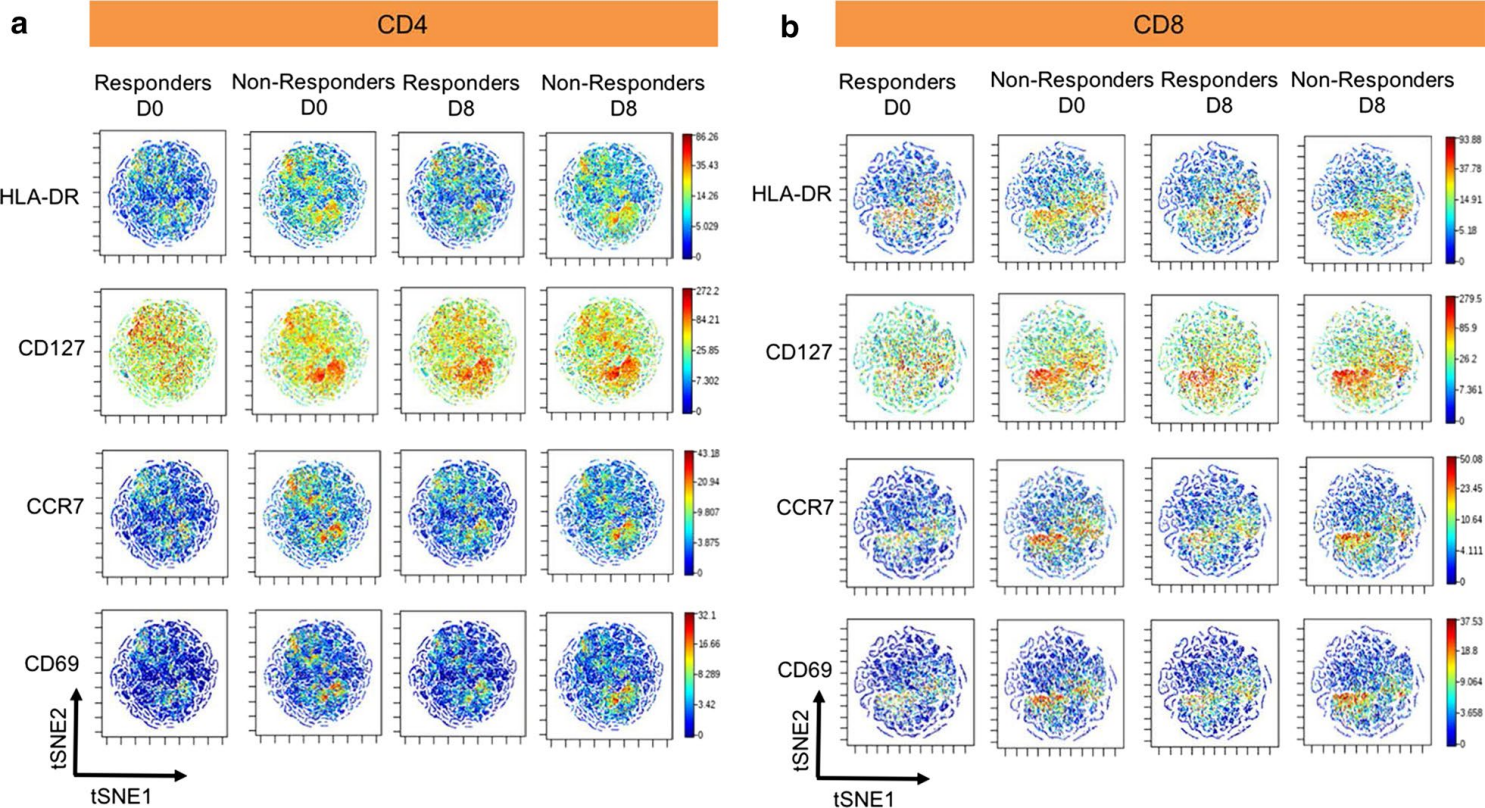
viSNE on antigen-specific cells finds differential markers. Boolean ‘OR’ gates for CD107a, IFNγ, TNFα and IL-4 from responders (n = 14) and non-responders (n = 5) were concatenated in Flowjo v10.1 for both CD4 and CD8 T cells after RSV (F) peptide stimulation at day 0 and day 8, and visualized in t-SNE space with viSNE software in Cytobank. viSNE analysis on antigen-specific T cells showed high expression of HLA-DR, CCR7, CD69 and CD127 in non-responders compared to responders at day 0 and day 8 in CD4 T cells (**a**). The same trend is seen in CD8 T cells (**b**) using this approach. Cells are colored by median intensity levels from high (red) to low (blue)

To determine differences in antigen-specific CD4^+^ and CD8^+^ T cells between responders and non-responders, we performed viSNE analysis. For each participant, boolean ‘OR’ gates on RSV specific CD107a^+^IFNγ^+^TNFα^+^IL4^+^CD4^+^ and CD8^+^ T cells pre- and post-vaccination (day 0 and day 8, respectively) were generated in flowjo v10.1. The individual fcs files for the boolean gates were then concatenated into single standard fcs files for responders and non-responders, resulting in a total of 4 concatenated files for each T cell population (Additional file 4). Using Cytobank software, the viSNE algorithm analysed ungated cell populations for equal number of events per time point for each T cell population (as described above in MM). viSNE plots are shown as two-dimensional scatter plots with the x- and y-axes identified by tSNE1 and tSNE2. Each dot on the plot represents a single cell positioned according to similarity in the high-dimensional space. For comparative purposes, viSNE on pre- and post-vaccinated days (Day 0 and Day 8) between responders and non-responders for each T cell population was performed in the same run. For both CD4^+^ (Fig. 6a) and CD8^+^ (Fig. 6b) T cell populations, viSNE analysis showed an increased expression of HLA-DR^+^, CD127^+^, CCR7^+^ and CD69^+^ cells in responders and non-responders post-vaccination (day 8) compared to baseline (day 0). Furthermore, the expression of CD4^+^ and CD8^+^ HLA-DR^+^, CD127^+^, CCR7^+^ and CD69^+^ cells was higher in the non-responders compared to the responders both pre- and post-vaccination (day 0 and day 8, respectively). Interestingly, for both CD4^+^ and CD8^+^ T cells, viSNE identified the same population of cells. The pattern of expression was similar but the intensity levels was different for all the markers. Again, the findings from PCA corroborate with some of the viSNE results, with CD4^+^HLA-DR^+^, CD4^+^CD69^+^, CD8^+^HLA-DR^+^, CD4^+^CCR7^+^ being positively co-related with the non-responders (Fig. 5b).

## Discussion

In this study, we successfully derived baseline cellular features from our CyTOF analyses that were associated with ELISPOT response to an experimental RSV vaccine. Notable among these features were high baseline levels of IFNγ-producing RSV-specific CD8^+^ T cells, which were associated with non-responder status. In the setting of influenza vaccination, a high baseline titer of hemagglutinin-inhibiting (HI) antibodies has been linked to lower fold-change in HI antibodies post-vaccination [26]. A similar relationship has been found for CD4^+^IFNγ^−^ producing T cells specific to influenza [27]. Our findings with this experimental RSV vaccine may be related, in that it may be more difficult to boost a pre-existing immune response with vaccination. As Falloon et al. concluded, a correlate of protection from RSV is yet unknown, and while both antibodies and T cells were induced by vaccination these did not provide protection [22].

We also saw an association of activated (HLA-DR^+^) CD4^+^ and CD8^+^ T cells with non-responder status. While the short term in vivo exposure to antigen should be too short to induce HLA-DR expression, these cells might indicate the level of chronic inflammation in these elderly subjects. Chronic inflammation in the elderly is considered a cause of immunologic aging [28]. Higher levels of inflammatory response transcripts have been linked with hypo responsiveness to hepatitis B vaccination [29].

The PCA-model revealed two study groups that formed two distinct clusters, a tight one composed of responders and a scattered one composed of non-responders. The results indicates that responders have a more homogenous and “healthy” phenotype before vaccination whereas non-responders do not. The different profiles explain why we see 9 variables associated with non-responders and only 4 variables associated with responders in the loading plot. Consequently, if a patient has increased levels of any of these 9 variables the outcome of the vaccine against RSV may be poor. A number of features from the PCA-model displayed excellent correlation with the viSNE analysis. High levels of CD4^+^HLA-DR^+^, CD4^+^CD69^+^, CD8^+^HLA-DR^+^, CD4^+^CCR7^+^ at baseline were associated with non-responders using both statistical methods. These variables were among the nine variables with the highest discriminatory power in the PCA model.

Surprisingly, our univariate analysis revealed that only CD8^+^IFNγ^+^ were statistically significant between responders and non-responders at baseline, although, a trend was observed at day 8. We could also see a minor increase for responders when comparing day 0 and day 29 for CD4^+^IFNγ^+^, CD4^+^IL4^+^ and CD8^+^ IL4^+^. Although this did not reach statistically significance, this was not seen for non-responders.

Among subjects with the highest responses (shown in the pie charts), a single cytokine seemed to dominate while the proportions were not greatly changed by vaccination, with the exception of IL-4 in the majority of pies and IL-17 as well as IFNγ in some pies. Interestingly, we could see a wide heterogeneity among the donors; different subjects have different cytokines that dominate in their immune profile, and in general, the same cytokine dominated before and after vaccination. The time point post vaccination were not optimal for studying innate immune cells, therefor we directed our attention to adaptive immune cells.

Possibly, the already activated state in non-responders makes it harder for their immune system to react again, as we previously discussed, and would benefit from a higher dose. If one could produce a more effective vaccine based on the immune profile of the subject perhaps immunosenescence would no longer be a determining factor for vaccine response. In addition, the huge economic cost for the society associated with elderly not responding to vaccines, resulting in increased medical care, increased use of medicines and lost time at work would benefit from vaccines that are designed after the immune profile of the subject at baseline. The elderly population [30] is increasing and finding vaccines and other pharmaceuticals suited for that expanding group is becoming substantially more important.

In this study, we demonstrate the advantage of combining vaccine studies with mass cytometry. It enables an understanding of the complex immune response that is difficult to obtain with other methods. Here, it specifically revealed how elderly responded to vaccines based on their baseline cellular features. With this high-dimensional technique, co-expression of cytokines can be studied in several cell types and subgroups in a single sample. As such, it helps us to paint a wider picture of the immune system. A challenging task is to process the large amounts of generated data and interpret it correctly. Therefore, it is essential to use multivariate analysis, e.g., PCA and viSNE, as we do in this study. As mass cytometry as well as more advanced multivariate methods are getting more available in vaccine research, we will be able to get a clearer picture and enhanced understanding of both immunosenescence and vaccine response.

## Conclusion

Our results show the impact of individual immune profiles on successful RSV-vaccine immune response. As our knowledge of the immune system increases, we believe this is a factor that needs to be accounted for when designing vaccines in the future. Taken together, our findings demonstrate the potential of CyTOF as a powerful technology that permits comprehensive profiling of immune components, thereby enabling prediction of responses to vaccines.

## Additional files

**Additional file 1.** Mock subtracted IFNγ responses at Day 1, at Day 8 undepleted, CD4 depleted and CD8 depleted.

**Additional file 2.** CyTOF workflow.

**Additional file 3.** Percentage of RSV specific CD3^+^CD4^+^ and CD3^+^CD8^+^ responses.

**Additional file 4.** Workflow to perform viSNE analysis on antigen-specific cells.

## Abbreviations

CyTOF: Cytometry by Time-of-Flight
RSV: respiratory syncytial virus
ELISPOT: The Enzyme-Linked ImmunoSpot
GLA-SE: Glucopyranosyl Lipid A in 2% stable emulsion
PBMC: peripheral blood mononuclear cells
SFC: spot forming cells
DMSO: dimethyl sulfoxide
SEB: *Staphylococcus aureus* enterotoxin B
PMA: Phorbol 12-myristate 13-acetate
PFA: paraformaldehyde
CMV: cytomegalovirus
DOTA: 1,4,7,10-tetraazacyclododecane-1,4,7,10-tetraacetic acid
PCA: principal components analysis
OPLS-DA: orthogonal partial least square-discriminant analysis
viSNE: visualization tool for high-dimensional single-cell data based on the t-Distributed Stochastic Neighbor Embedding (t-SNE) algorithm
HI: hemagglutin-inhibiting
