# Supplementary figures and images for: Baseline immune profile by CyTOF can predict response to an investigational adjuvanted vaccine in elderly adults
Source: J Transl Med. 2018 Jun 5;16:153. doi: 10.1186/s12967-018-1528-1 (PMC5987461; doi:10.1186/s12967-018-1528-1)

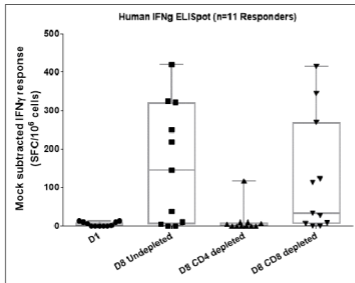

Supplement: Supplementary file 1 — Additional file 1. Mock subtracted IFNγ responses at Day 1, at Day 8 undepleted, CD4 depleted and CD8 depleted. [file 12967_2018_1528_MOESM1_ESM.pdf]

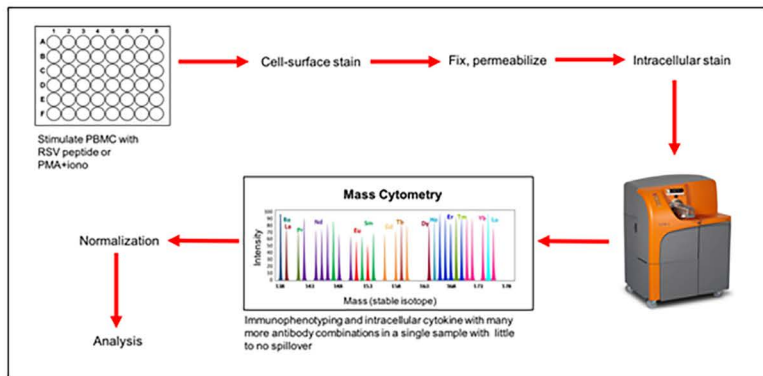

Supplement: Supplementary file 2 — Additional file 2. CyTOF workflow. [file 12967_2018_1528_MOESM2_ESM.pdf]

**A****RSV**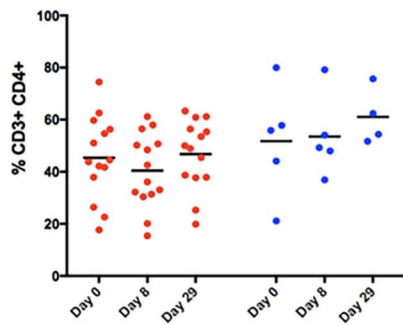**B****RSV**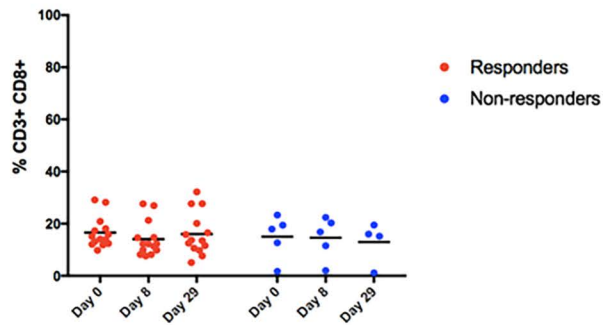

Supplement: Supplementary file 3 — Additional file 3. Percentage of RSV specific CD3+CD4+ and CD3+CD8+ responses. [file 12967_2018_1528_MOESM3_ESM.pdf]
