# Supplementary material for: Baseline immune profile by CyTOF can predict response to an investigational adjuvanted vaccine in elderly adults
Source: J Transl Med. 2018 Jun 5;16:153. doi: 10.1186/s12967-018-1528-1 (PMC5987461; doi:10.1186/s12967-018-1528-1)
Supplement: Supplementary file 4 — Additional file 4. Workflow to perform viSNE analysis on antigen-specific cells. [file 12967_2018_1528_MOESM4_ESM.pdf]

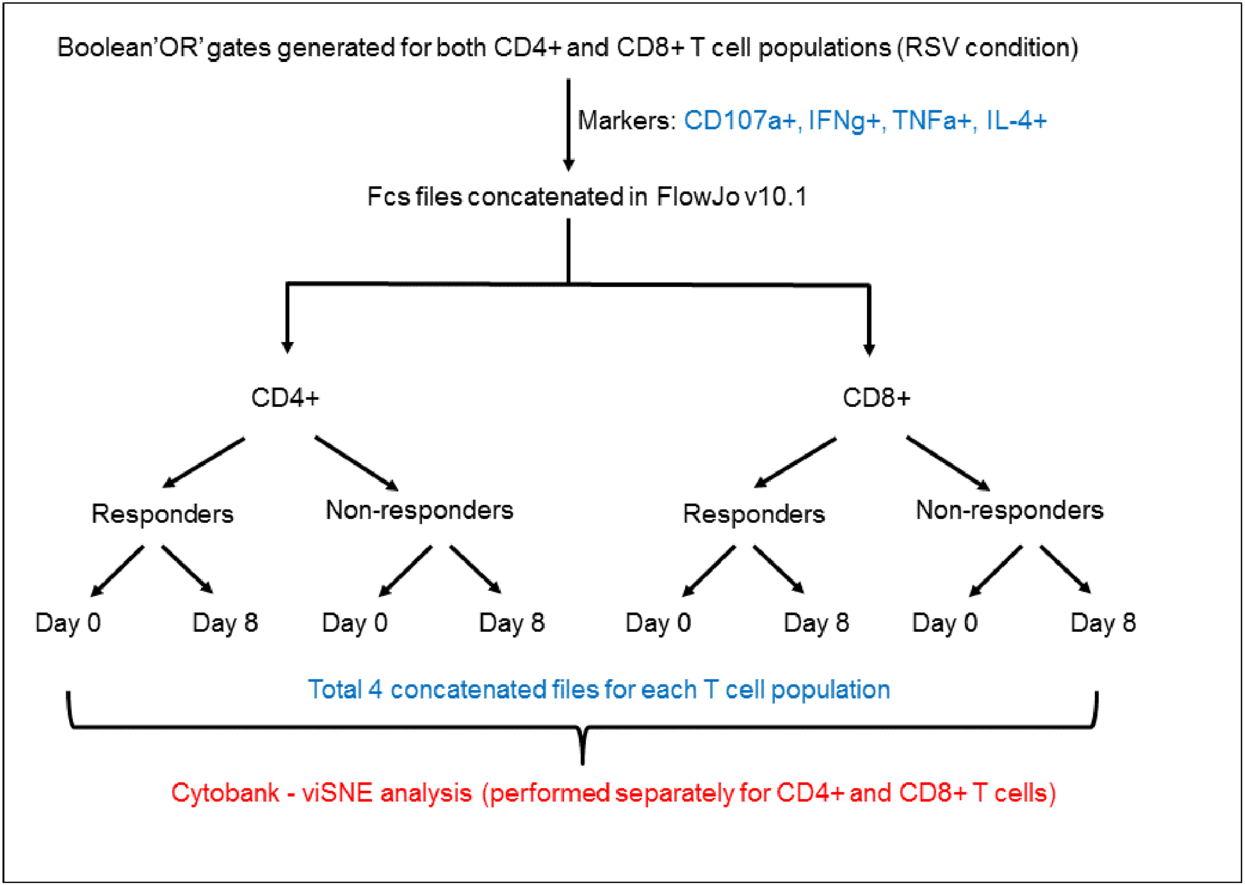
Boolean'OR' gates generated for both CD4+ and CD8+ T cell populations (RSV condition)
Markers: CD107a+, IFNg+, TNFa+, IL-4+
Fcs files concatenated in FlowJo v10.1
CD4+
CD8+
Responders
Non-responders
Responders
Non-responders
Day 0
Day 8
Day 0
Day 8
Day 0
Day 8
Day 0
Day 8
Total 4 concatenated files for each T cell population
Cytobank - viSNE analysis (performed separately for CD4+ and CD8+ T cells)
